# Proton magnetic resonance spectroscopy (^1^H-MRS) of the brain in patients with tick-borne encephalitis

**DOI:** 10.1038/s41598-019-39352-6

**Published:** 2019-02-26

**Authors:** Radosław Zawadzki, Bożena Kubas, Marcin Hładuński, Olga Zajkowska, Joanna Zajkowska, Dorota Jurgilewicz, Adam Garkowski, Sławomir Pancewicz, Urszula Łebkowska

**Affiliations:** 1 Department of Radiology, Medical University of Bialystok, Bialystok, Poland; 2 Independent Department, Laboratory of Molecular Imaging, Medical University of Bialystok, Bialystok, Poland; 3 Faculty of Applied Informatics and Mathematics, Warsaw University of Life Sciences SGGW, Warsaw, Poland; 4 Department of Infectious Diseases and Neuroinfections, Medical University of Bialystok, Bialystok, Poland

## Abstract

Tick-borne encephalitis (TBE) is a disease caused by a tick-borne encephalitis virus (TBEV) belonging to the *Flaviviridae* family. The aforementioned virus is transmitted by the bite of infected ticks. In the recent years, TBEV has become a serious public health problem with a steady increase in its incidence, mainly due to the climate changes and spreading the infected ticks into new territories. The standard protocol of TBE diagnosis involves the serological laboratory test with a minor role of imaging techniques such as magnetic resonance imaging. Long-term complications affecting patients daily activities are reported in about 40–50% of the cases. However, no changes are revealed in the laboratory tests or the imaging examination. The development of new imaging techniques such as proton magnetic resonance spectroscopy (^1^H-MRS) can broaden the knowledge about TBE, contributing to its prevention. The aim of this study was to assess the usefulness of ^1^H-MRS of the brain in patients with TBE. Compared to controls, a statistically significant decrease in the N-acetylaspartate /creatine ratio was found bilaterally in the right and left thalamus as well as a statistically significant increase in the choline/creatine ratio in the right and left thalamus.

## Introduction

Tick-borne encephalitis virus (TBEV) poses a serious public health problem, especially, in the endemic areas of Central, Eastern and Northern Europe, in northern China, Mongolia, South Korea and in the Russian Federation. TBEV belongs to the family *Flaviviridae*, genus *Flavivirus*, is transmitted through a tick-bite, or is connected with the consumption of non-pasteurized milk from infected livestock^1^. There are three subtypes of the virus causing a human disease: the European subtype, the Far-Eastern subtype and the Siberian subtype^2^ including mainly *Ixodes ricinus* in Europe, *I. persulcatus* predominating in the Siberian subtypes and *I. ovatus* of Far-Eastern TBEV subtype in Japan^3–5^. The vectors of TBEV are infected ticks. Most cases of TBE occur in the period between April and November with the peak of the tick activity which corresponds with warming of the climate during spring, summer and autumn months and human outdoor activity^6–8^.

The disease has a biphasic course with an incubation period varying from 2–28 days (usually 7–14 days), and shorter incubation periods after milk-borne exposure with the first phase lasting approximately 1–8 days. In this phase, the patient has nonspecific flu-like symptoms^9,10^. After about 8 days of remission, in 30–50% of the cases, the second neurological phase begins with symptoms in the form of meningitis (50% of the adult cases), meningoencephalitis (about 40% of the adult patients), myelitis (4–15%)^11,12^. It is important to note that about 40–50% of the patients who underwent TBE can develop a post-encephalic syndrome which results in permanent CNS function impairment in the form of cognitive disorders and neuropsychiatric complaints^10,13–15^.

Currently the diagnosis of TBE is based on the laboratory serological tests with a subsidiary role of magnetic resonance (MR) imaging techniques and a minor role of computed tomography (CT) imaging. In the first viral phase of the disease, leukopenia and/or thrombocytopenia can be observed, whilst a mildly elevated number of leukocytes in the second encephalic phase^16^. The key role in diagnosing TBE plays determination of TBEV specific IgM and IgG serum antibodies obtained by means of a highly specific immunosorbent assay (ELISA), while polymerase chain reaction (PCR) helps in the virus detection only in the first viral phase of the infection. Additionally, the cerebrospinal fluid (CSF) analysis may reveal pleocytosis.

Due to the limited spatial resolution, CT is considered to be less sensitive than MR and has an auxiliary role before the lumbar puncture to exclude brain oedema. To this time there has been a limited number of reports involving macroscopic CNS changes exhibiting hypodense areas in the CNS^17–19^.

MR imaging can be helpful in diagnosing inflammatory morphological changes in the CNS. The optimal time of the MR scan correlates with the best detectability of the IgM and IgG antibodies in the CSF at around the tenth day of the infection^20^. A standard MR examination can show lesions in the thalamus, basal ganglia, internal capsule, splenium, cerebellum, peduncles and brainstem, while a small number of patients with encephalomyeloradiculitis, encephalomyelitis, isolated radiculitis, myeloradiculitis had also documented spinal MRI changes^4,21–34^.

The purpose of this study was to evaluate whether patients with TBE may develop brain metabolite alterations within the brain regions without any macroscopic changes on the conventional MR examination, especially, considering the fact that in about 40–50% of the cases, long-term complications affecting daily activity are reported without any changes in the laboratory test or the imaging examination. It is important to note that to this date and to our knowledge no major studies describing the features of TBE in 1H-MRS have been conducted in people. We hypothesized that since TBEV infects the brain, it may lead to alterations in the cerebral metabolism measured by ^1^H-MRS.

## Materials and Methods

### Study subjects and protocol of proton magnetic resonance spectroscopy

The present study was carried out in 25 patients diagnosed with TBE and 25 healthy controls. Only patients with meningoencephalitis and meningoencephalomyelitis, were included in the study excluding those with meningitis, since they presented no neurological symptoms,thus no 1H-MRS abnormalities were expected. The clinical course of the disease, age and sex distribution are presented in Table 1. No statistically significant difference was found in age and gender distribution between the two groups. The inclusion criteria were based on the European Centre for Disease Prevention and Control (ECDC) guidelines and consisted of a typical two phase clinical course of TBE, detection of IgM and/or IgG antibodies in blood serum and CSF by ELISA as well as granulacytosis in the CSF examination.

**Table 1:** Epidemiological and clinical characteristics of patients with tick-borne encephalitis.

Characteristic	No. of Patients (%)
Mean age, years (range)	43.6 (22–64)
Male, sex	15 (60%)
Female, sex	10 (40%)
History of tick bite	9 (36%)
**Clinical course of TBE:**	
Meningitis	0
Meningoencephalitis*	22 (88%)
Meningoencephalomyelitis*	3 (12%)

*These patients had consciousness disturbances and/or focal neurological symptoms. Patients with epileptic seizures were not included in the study, because they can change ^1^H-MRS results.

### Protocol of MRI and proton magnetic resonance spectroscopy (^1^H-MRS)

All patients underwent a routine MRI protocol with a single 3.0 Tesla MRI scanner (Biograph mMR, Siemens Healthineers, Erlangen, Germany), using a 16-channel head matrix coil.

Scan was performed between 10^th^ and 14^th^ day of the disease. Routine imaging was carried out prior to spectroscopy to exclude any structural abnormalities and included T1W (TR, 180 ms; TE, 2,5 ms; section thickness, 4.0 mm; intersection gap, 1,0 mm; 27 axial slices; field of view [FOV], 220 × 185 mm; and matrix size, 320 × 320), T2W imaging (TR, 3300 ms; TE, 107 ms; section thickness, 4.0 mm; intersection gap, 1 mm; 30 axial slices; field of view [FOV], 220 × 185 mm; and matrix size, 512 × 512), fluid- attenuated inversion recovery (FLAIR) (TR, 9000 ms; TE, 94 ms; section thickness, 4.0 mm; intersection gap, 1,0 mm; 27 axial slices; field of view [FOV], 240 × 195 mm; and matrix size, 256 × 256), and diffusion weighted images (DWI) (TR, 870 ms; TE, 82 ms; section thickness, 4,0 mm; intersection gap, 1,0 mm; 27 axial slices; FOV 240 × 240 mm; and matrix size 132 × 132; b-value 0, 500, 1000, 1500).

**Figure 1:**
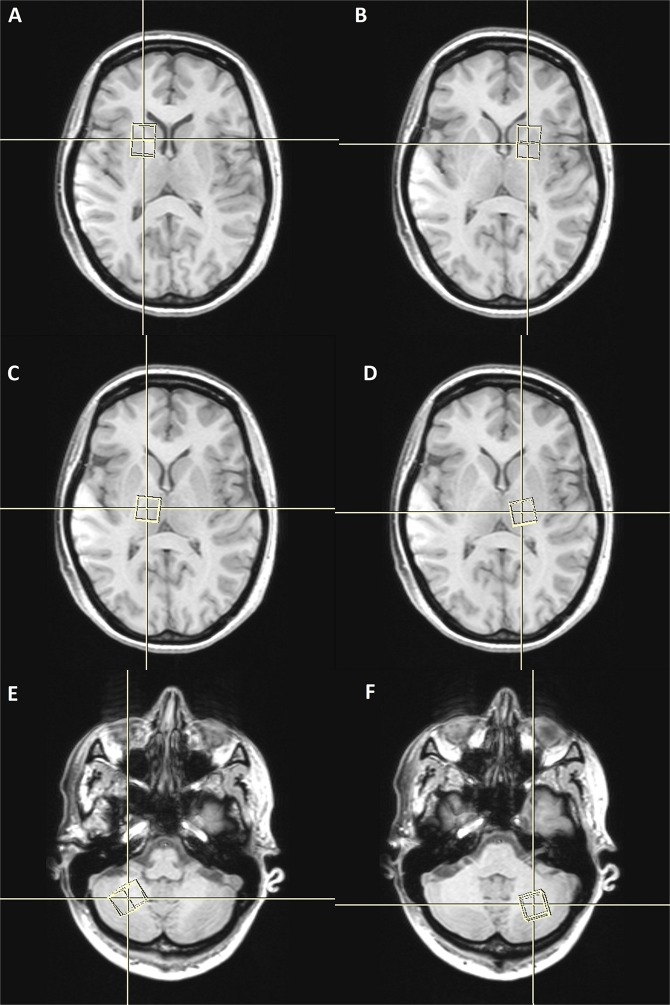
Spatial localization of the VOI in the in the basal ganglia (**A**,**B**) thalami (**C**,**D**) hemispheres of cerebellum (**E**,**F**).

**Figure 2:**
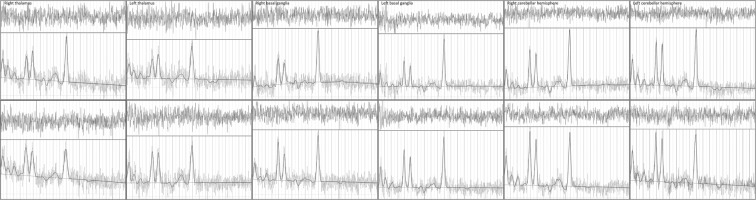
Sample spectra for healthy controls (upper panel) and patients with TBE (lower panel) from the six volumes of interest.

The preparation phase of ^1^H-MRS included automatic procedures for water suppression, shimming, and tuning of the radiofrequency and gradient system and an acquisition without water suppression for correction of magnetic field distortions. ^1^H-MRS planning was acquired on T1W sequences (TR; 18 ms; TE; 4,97 ms, section thickness 1 mm; intersection gap 0,2 mm; 160 saggital slices; FOV [230 × 230]; and matrix size 256 × 256) from this sequence, coronal and axial planes were reconstructed with the same technical parameters. ^1^H-MRS examinations were performed with the following acquisition parameters: (TR/TE: 2000/135 ms, bandwidth 1200 Hz, number of excitation, 128). A point-resolved spectroscopy sequence (PRESS) was used for the rectangular volume of interest (VOI) selection. Single VOIs were manually located bilaterally within the normal-appearing brain tissue in the basal ganglia, thalami and in the hemispheres of the cerebellum with the size of VOI of 15 × 15 × 15 mm (Fig. 1). VOIs were positioned aiming to cover the regions that had been investigated for the greatest viral accumulation shown in the previous studies^35^. The localization of VOIs was confirmed by three orthogonal MR images in the axial, sagittal, and coronal planes. Metabolite ratios were calculated based on the peak integrals of N-acetylaspartate (NAA, 2.01 ppm), creatine (Cr, 3.03), choline-containing compounds, (Cho, 3.23 ppm). The metabolite concentrations (Fig. 2) were analysed using LCmodel (Provencher, 1993) quantification algorithm^36^.

The MR images were interpreted by a senior MR radiologists (with 20 years of experience in MRI).

### Statistical analysis

The significance of differences in the metabolite ratios between TBE and control group were estimated with the unpaired Welch’s t test with unequal variances. Since the total of TBE patients and control group is limited, the p-values were bootstrapped for the two tailed t test with 10000 replicates. A value of p < 0.05 was considered statistically significant. The robustness of the results was confirmed by means of the nonparametric methods (Kruskal-Wallis test, Wilcoxon rank sum test). The analysis was performed with Stata 15 software.

### Ethical approval and informed consent

The study was approved by the ethics committee of the Medical University of Bialystok (approval no. R-I-002/340/2015) and was conducted according to the principles expressed in the Declaration of Helsinki. A written informed consent was obtained from all patients.

## Results

The NAA/Cr ratio was significantly lower in the left and right thalamus (bootstrapped p = 0.000 and p = 0.001, respectively). Additionally, the difference was also found in the right basal ganglia confirmed by both the one-sided t-test and nonparametric methods. A statistically significant increase in the Cho/Cr ratio was observed in the left thalamus and left cerebellar hemisphere (bootstrapped p = 0.007 and p = 0.016 respectively), as shown in Table 2. There was no statistically significant brain metabolite changes in other study areas.

**Table 2:** 1H-MRS data for patients and control group.

VOI location	NAA/Cr	Control group	Cho/Cr	Control group
	Patients		Patients	
Right thalamus	1,863 ± 0,246*	2,170 ± 0,270	0,358 ± 0,038	0,337 ± 0,062
Left thalamus	1,988 ± 0,365*	2,284 ± 0,234	0,365 ± 0,051*	0,332 ± 0,034
Right basal ganglia	1,389 ± 0,171*	1,631 ± 0,644	0,288 ± 0,044	0,311 ± 0,145
Left basal ganglia	1,441 ± 0,173	1,442 ± 0,207	0,279 ± 0,035	0,273 ± 0,062
Right cerebellar hemisphere	1,563 ± 0,307	1,545 ± 0,196	0,326 ± 0,065	0,311 ± 0,039
Left cerebellar hemisphere	1,577 ± 0,223	1,489 ± 0,220	0,342 ± 0,048*	0,312 ± 0,038

*p < 0.05.

The examination was performed between 10th and 14th day of the disease. No macroscopic CNS lesions were found in the study group after MRI examination, apart from one patient presenting the dimmed nonspecific lesion observed in the left frontal lobe and bilateral diffused hyperintense lesions in the posterior horns of the lateral ventricles.

## Discussion

Findings presented in our study suggest that the TBEV infection may lead to metabolite alterations in the CNS, which can be in turn associated with the generation and persistency of psychiatric symptoms. The metabolite changes in the CNS of patients affected by TBEV could explain their clinical state, and can possibly affect their further loss of life quality. To the best of our knowledge, no ^1^H-MRS studies carried out in patients with TBE have been reported. Only one report on the examination performed in veterinary medicine involving dog models has been found^37^. The authors of that paper showed changes from a mild to moderate decrease in NAA and Cr peaks, and a mild increase in glutamate/glutamine peaks. It is worth mentioning that the TBEV infection is characterised by the distribution of the flavivirus (FV) attachment factors and receptors (GAGs, HSPGs, CD81, claudins, DC-SIGN) promoting FV entry into the neurons, glia and non-CNS tissues, which facilitates FV entry into these cells via a multi-step process, sometimes involving one or more of these attachment factors^38^. This leads to the rapid microglial/macrophage activation, release of soluble inflammatory mediators and over expression of MHC class I molecules as well as the recruitment of cytotoxic T cell, leading to three possible mechanisms of tissue destruction: by direct neuronal injury, by induction of an inflammatory response inducing the neuronal death, or a combination of these two mechanisms. The study revealed the following principal findings: NAA/Cr levels decreased bilaterally in the thalamus and the right-sided basal ganglia, while Cho/Cr levels increased in the left thalamus and left cerebellar hemisphere^39,40^. NAA is a marker of neuronal density and integrity, so the decreased values of NAA in the examined area prove that the axonal injury has taken place, even without any macroscopic changes in a routine MR examination both in the thalami and in the basal ganglia. A statistically significant increase in Cho/Cr was observed in the left thalamus and left cerebellar hemisphere. Choline as one of the components of the phospholipid metabolism of the cell membrane. Thus, disturbances in the Cho levels can cause problems with transporting and cell proliferation within the area affected. Moreover, acetylocholine, a precursor of choline, is a neurotransmitter which plays a major role in expressing emotions, whereas fluctuations in the levels of choline and compounds containing choline can explain emotional disturbances. No correlations were found between the fluctuations of metabolite levels and macroscopic changes in the study group. Only one patient had a subcortical nonspecific dimmed lesion in the left frontal lobe and bilateral diffused hyperintense lesions in the posterior horns of the lateral ventricles. This can prove that metabolite level changes occur first before macroscopic changes can be seen.

## Conclusions

The reduction of NAA/Cr and increase Cho/Cr ratio observed in patients that suffered from TBE can suggest a direct neurotropic activity of the TBEV. Our study has proved that ^1^H-MRS can be a sensitive tool for assessing brain metabolite changes in patients with TBE.
